# Pressure increases PD-L1 expression in A549 lung adenocarcinoma cells and causes resistance to anti-ROR1 CAR T cell-mediated cytotoxicity

**DOI:** 10.1038/s41598-022-10905-6

**Published:** 2022-04-28

**Authors:** Zhenglin Ou, Xiaolin Dou, Neng Tang, Guodong Liu

**Affiliations:** 1 Department of General Surgery, Xiangya Hospital Central South University, Xiangya Road 87#, Changsha, 410008 Hunan China; 2 National Clinical Research Center for Geriatric Disorders, Central South University, Changsha, Hunan China; 3 Department of Geriatric Surgery, Xiangya Hospital Central South University, Xiangya Road 87#, Changsha, 410008 Hunan China

**Keywords:** Cancer, Cell biology, Immunology

## Abstract

Due to the abnormal vasculation and proliferation, the tumor microenvironment is hypoxic, lacking nutrients, and under high interstitial pressure. Compared to oxygen and nutrients, the effect of pressure on cancer biology remains poorly studied. Here we constructed αROR1-CAR T cells and co-cultured with A549 cells with and without elevated pressure. We then measured apoptosis and cell death by flow cytometry and luciferase activity. We also measured cytokine (IL-2, IFN-γ, and TNF-α) release by ELISA. The results show that pressure-preconditioned A549 cells are much resistant to αROR1-CAR T cell-mediated cytotoxicity. Pressure preconditioning does not appear to affect the expression of αROR1-CAR or cytokine production. However, pressure preconditioning upregulates PD-L1 expression in A549 cells and decreases cytokine release from αROR1-CAR T cells. In addition, Pembrolizumab and Cemiplimab that block PD-1::PD-L1 interaction increase the cytokine production in αROR1-CAR T cells, increase the apoptotic cell death in A549 cells, and improve the αROR1-CAR T-mediated cytotoxicity. In xenograft mice, pressure preconditioning increases tumorigenesis of A549 cells, which can be blocked by a combined therapy using Pembrolizumab and αROR1-CAR T cells. Together, our studies suggest that elevated pressure in the tumor microenvironment could blunt the T cell therapy by upregulating PD-L1 expression, which could be overcome by combining CAR T therapy with immune checkpoint inhibitors.

## Introduction

Chimeric antigen receptor (CAR) T cells are therapeutic T cells that have been genetically engineered to express receptors that recognize specific cancer antigens^1,2^. In cancer biology, multiple mechanisms can cause cancer cells to escape immune surveillance. Among them, a major issue is the thymic elimination of high‐affinity T‐cell receptors (TCRs), which leads to relatively low‐affinity TCRs against “self” antigens including some cancer antigens^3^. CARs are devised to re-direct T cells to recognize and destroy cancer cells as “foreign” agents. In a typical CAR, the single-chain variable fragment (ScFv) of a cancer antigen-recognizing antibody is fused to the intracellular fragment of the TCR containing the signaling domains CD3ζ and co-stimulatory domains CD28 and 4-1BB^4^. The CARs-encoding DNA is generally delivered into a patient’s T cells in vitro through virus transduction. The T cells expressing the CAR (CAR T cells) are then infused back into the patient’s circulatory system. Due to the high affinity of the ScFv to cancer antigens, CAR T cells can recognize and destroy cancer cells that have escaped the host immune surveillance.

In recent years, CAR T‐cell therapies for B cell malignancies have seen dramatic clinical responses with a high rate of complete remission^5^. However, their applications in solid tumors remain challenging^1^. Different from leukemia, solid tumors have densely packed cancer cells lacking sufficient nutrients and oxygen and under differential interstitial fluid pressure^6,7^. The tumor interstitial fluid pressure (TIFP) is caused by rapid accumulation of cancer cell mass, overly stimulated angiogenesis, and interstitial fibrosis^8^. TIFP is estimated to be 5–40 mmHg higher than normal tissues^9–11^. High TIFP can lead to inadequate tumor perfusion resulting from missing lymphatic vessels, a leaky and immature tumor vasculature, which in turn cause inadequate lymphocyte infiltration and drug delivery^8,12^. High TIFP has also been shown to rewire the global gene transcription programs related to extracellular matrix and stress resistance, leading to growth advantage under the adverse tumor microenvironment^13,14^. Consistently, high TIFP is correlated with poor prognosis, metastasis and resistance to radiation therapy, and chemotherapy in many types of cancer^15–19^. Lowering TIFP decreases cancerous proliferation and shows promising results in improving chemotherapy^20,21^. However, in contrast to hypoxia and nutrient limitation, how TIFP induces resistance to cancer therapy remains poorly understood.

PD-1 and PD-L1 are immune checkpoint proteins that mediate cytotoxicity of cancer immune therapy^22,23^. PD-1 is expressed by activated T lymphocytes and other immune cells on the cell membrane^24^. Tumor cells broadly express PD-1 ligands PD-L1 and PD-L2, which interact with PD-1, therefore inhibiting lymphocyte-mediated cytotoxicity. High PD-L1 expression predicts poor survival in many types of cancer^25,26^. Anti-PD-1 antibodies such as Pembrolizumab and Cemiplimab block the interaction between PD-1 and PD-L1, therefore improving the cytotoxicity of T cells^27^. Pembrolizumab and Cemiplimab have been approved by FDA as second-line treatment for leukemias refractory to chemotherapy. In the solid tumor microenvironment, hypoxia increases the expression of PD-L1 in various tumor cells, leading to decreased T cell cytotoxicity and increased immune escape^28^. Whether elevated pressure in solid tumors could alter immune checkpoint protein has not been reported.

In this study, we investigated if elevated pressure could affect αROR1-CAR T cell-mediated cytotoxicity in lung cancer cell line A549 and whether immune checkpoint PD-L1 was involved. Our results showed that applying additional 100 mmHg to A549 cells reduced the potential of αROR1-CAR T cells to kill A549 cancer cells. We found that the reduced cytotoxicity was attributed to increased PD-L1 expression in A549 cells in response to elevated pressure. Blocking PD-1 and PD-L1 interaction by Pembrolizumab or Cemiplimab increased cytokine release and enhanced αROR1-CAR T cell-mediated cytotoxicity. We further confirmed these results in xenograft mice. Our study could have significant implications for the clinical treatment of solid tumors.

## Results

### Elevated pressure reduced αROR1-CAR T cell-mediated cytotoxicity in A549 lung cancer cells

**Figure 1:**
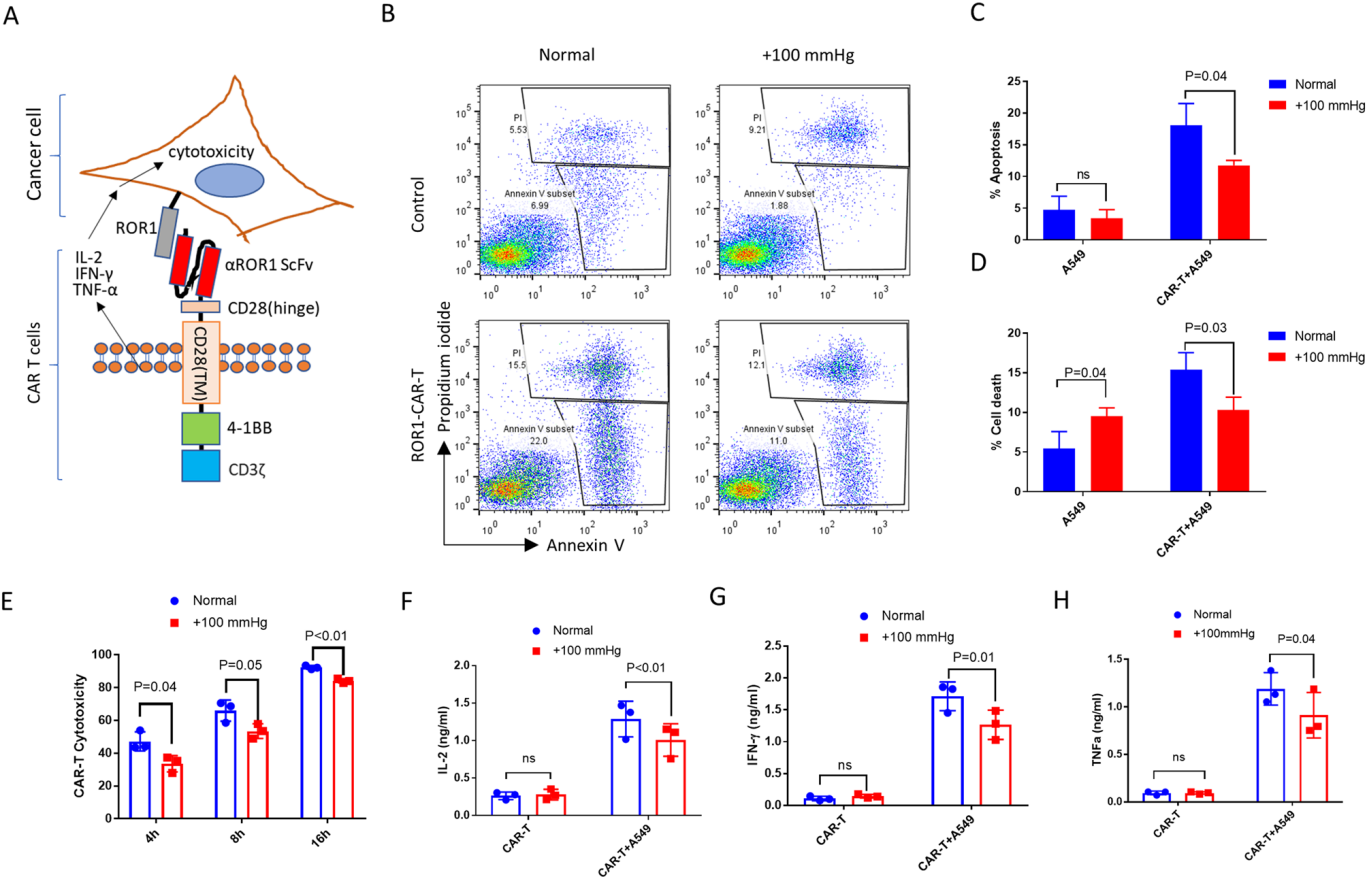
Elevated pressure reduced αROR1-CAR T cell-mediated cytotoxicity in A549 lung cancer cells. (**A**) A schematic showing the αROR1-CAR T cells in activating cytotoxicity in cancer cells. The structure of the αROR1-CAR is composed of an αROR1 ScFv, CD28 transmembrane domain, a 4-1BB co-stimulatory domain, and a CD3ζ signaling domain. (**B**) A549 cells maintained in elevated pressure (+ 100 mmHg) for at least 7 days (2 passages) were co-cultured without or with αROR1-CAR T cells at 1:10 ratio in a pressured incubator for 4 h. A549 cells were gated according to size (Fig. S4) and apoptotic (Annexin V+ Propidium iodide−) and dead (Annexin V + Propidium iodide +) cells were quantified by flow cytometry. (**C**,**D**) Quantification and statistical analysis of apoptosis and cell death by 3 biological repeats. P indicates P values and ns indicates no significance. (**E**) Cytotoxicity assay was performed as in (**A**) except using cells stably expressing fly luciferase (A549-Red-Fluc) as a convenient readout. Cell viability was measured directly through luciferase activity and the reads were converted to cytotoxicity as mentioned in “Methods”. (**F**–**H**) Cytokines (IL-2, IFN-γ, and TNF-α) in the medium after 16 h of co-culture were measured by ELISA.

We have been interested in improving CAR T cells for solid cancer treatment. We constructed an αROR1-CAR using the highly specific ScFv of a rabbit anti-human ROR1 monoclonal antibody (mAb) published before^29^ (see details in “Methods”). ROR1 was broadly expressed on many tumor cells, including lung adenocarcinomas cell A549^30,31^. αROR1-CAR T cells can be activated by ROR1-ScFv conjugation, releasing cytokines including IL-2, IFN-γ, and TNF-α and inducing cytotoxicity in cancer cells (Fig. 1A). To evaluate whether pressure would alter the cytotoxicity of αROR1-CAR T cells to A549 cells, we added freshly prepared αROR1-CAR T cells to A549 cells that have been cultured in normal or elevated pressure (+ 100 mmHg) for 2 passages (~ 7 days). After 4 h of co-culture, the αROR1-CAR T cells effectively induced apoptotic cell death in A549 cells (Fig. 1B–D). Importantly, both apoptosis and cell death were reduced by elevated pressure. Elevated pressure did not affect apoptosis of A549 cells alone; instead, it slightly increased the cell death in several experimental repeats (Fig. 1D).

To conveniently evaluate the cytotoxicity of our αROR1-CAR T cells, we incubated A549-Red-Fluc cells stably expressing fly luciferase with CAR-T cells for 4, 8, and 16 h, and measured cell viability through detecting luciferase activity. Consistently with the flow cytometry results, elevated pressure slightly reduced the cytotoxicity of αROR1-CAR T cells to A549-Red-Fluc cells (Fig. 1E). We measured the common cytokines involved in T cell proliferation and cytotoxicity including IL-2, IFN-γ, and TNF-α. The results showed that elevated pressure reduced the secretion of these cytokines (Fig. 1F–H). Similar results were obtained by using PBMCs (peripheral blood mononuclear cells) from two other donors.

### Pressure preconditioning did not affect the cytotoxicity of αROR1-CAR T cells

**Figure 2:**
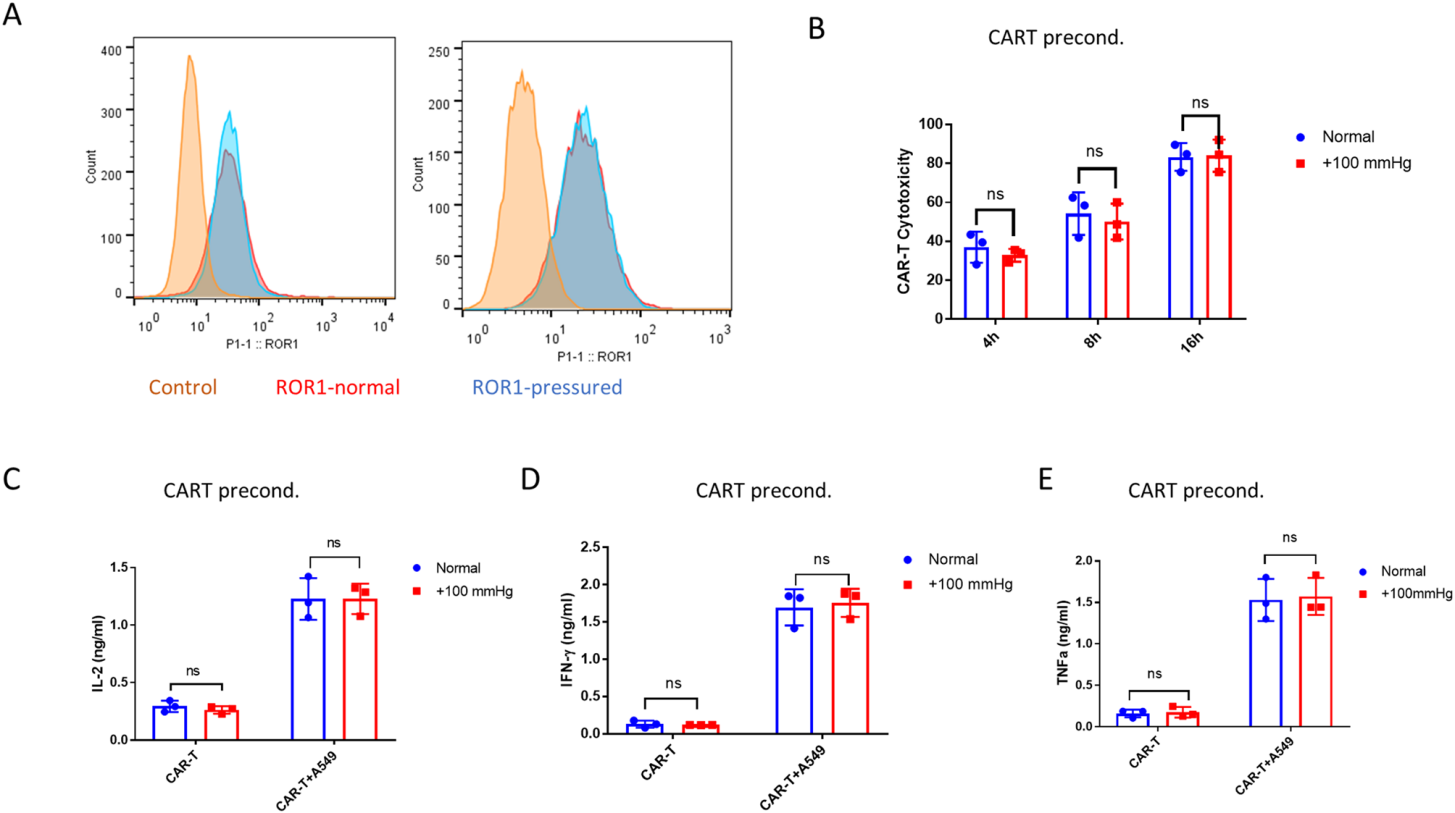
Pressure preconditioning did not affect the cytotoxicity of αROR1-CAR T cells. (**A**) Pressure preconditioning did not affect αROR1-CAR expression. αROR1-CAR T cells were maintained in a pressurized incubator (+ 100 mmHg) for 24 h and the expression of αROR1-CAR was detected by flow cytometry. Shown are data from 2 biological repeats. (**B**) αROR1-CAR T cells preconditioned in pressurized incubator (+ 100 mmHg) for 24 h were co-cultured with non-preconditioned A549-Red-Fluc cells under normal conditions. Viability at indicated time points was measured by luciferase activity and converted to cytotoxicity. (**C**–**E**) Cytokines (IL-2, IFN-γ, and TNF-α) in the medium after 16 h of co-culture shown in (**B**) were measured by ELISA.

Because both CAR T cells and cancer cells were under elevated pressure in cytotoxicity assay, the reduced cytotoxicity could be attributed to either αROR1-CAR T, A549, or both. We first tested if preconditioning of αROR1-CAR T cells under elevated pressure (+ 100 mmHg) for 24 h could result in reduced cytotoxicity. We verified that the αROR1-CAR expression on T cell surface was not changed by 24 h of pressurized culture (Fig. 2A). We then preconditioned αROR1-CAR T cells with + 100 mmHg for 24 h and co-cultured with A549-Red-Fluc cells under normal conditions for 16 h. Interestingly, 24-h pressure preconditioning did not significantly alter CAR-T cytotoxicity (Fig. 2B). Cytokines in the co-culture medium were evaluated by enzyme-linked immunosorbent assay (ELISA). Consistent with cytotoxicity assay, IL-2, IFN-γ, and TNF-α were not changed by pressure preconditioning of αROR1-CAR T cells (Fig. 2C–E).

### Pressure preconditioning A549 cells increased PD-L1 expression and caused resistance to αROR1-CAR T cell treatment

**Figure 3:**
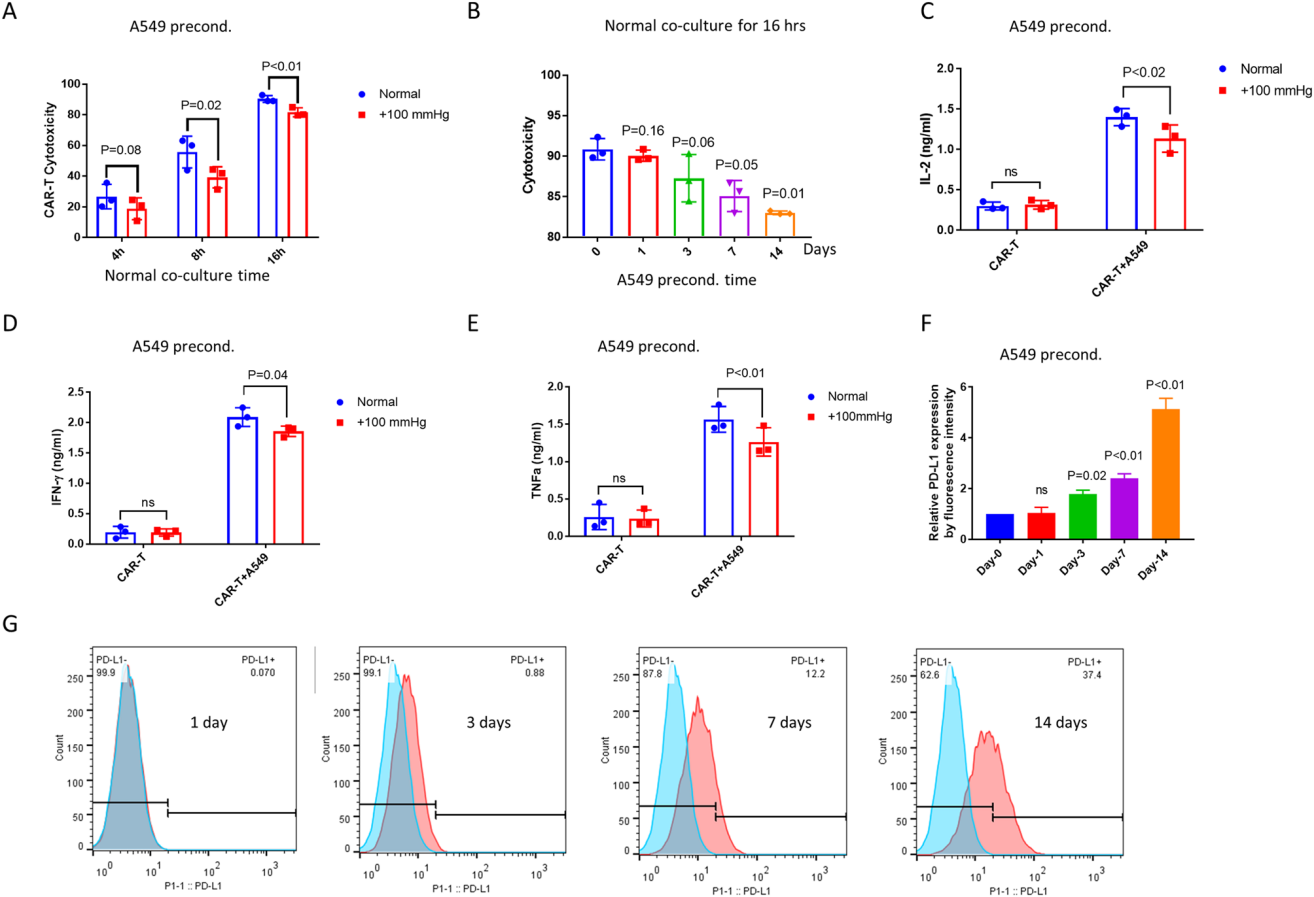
Pressure preconditioning A549 cells increased PD-L1 expression and caused resistance to αROR1-CAR T cell treatment. (**A**) A549-Red-Fluc cells preconditioned in pressurized incubator (+ 100 mmHg) for 7 days were co-cultured with non-preconditioned αROR1-CAR T cells under normal conditions. Cytotoxicity at indicated time points was calculated based on luciferase activity. (**B**) Pressure preconditioning decreased αROR1-CAR T cell cytotoxicity in a time-dependent manner. A549-Red-Fluc cells were preconditioned for indicated time and co-cultured with non-preconditioned αROR1-CAR T cells under normal conditions for 16 h. αROR1-CAR T cytotoxicity were calculated by luciferase activity. (**C–E**) Cytokines (IL-2, IFN-γ, and TNF-α) in the medium of co-culture shown in (**B**) were measured by ELISA. (**F**) PD-L1 expression in A549 cells was elevated by pressure. Data shown the quantification and statistical analysis of 2 biological repeats. (**G**) A typical experiment shown in (**F**). A549 cells were preconditioned for 1, 3, 7 and 14 days. Expression of PD-L1 was measured by flow cytometry and presented in histogram (red). Non-preconditioned A549 cells were used as controls (blue).

We next asked if A549 cells could become more resistant to CAR-T cells under elevated pressure. To mimic chronic pressure in solid tumors, we have maintained A549 cells in + 100 mmHg for at least 2 passages (~ 7 days). We pressure-preconditioned A549 cells for 7 days but co-cultured with non-pressurized αROR1-CAR T cells for 4, 8, and 16 h. Indeed, pressure preconditioning of A549 cells slightly increased the resistance to αROR1-CAR T cell cytotoxicity (Fig. 3A). To further confirm this result, we cultured A549 cells under + 100 mmHg pressure for 1, 3, 7, and 14 days and examined the cytotoxicity after incubating with CAR-T cells for 16 h. We found a time-dependent resistance of A549 cells to CAR-T cytotoxicity (Fig. 3B). 14-day and 7-day preconditioning caused significant resistance to αROR1-CAR T cells; 3-day preconditioning followed the same trend (*p* = 0.06); 1-day preconditioning showed no discernible effect. Consistently, the cytokine release experiment confirmed that pressure-preconditioning over 7 days suppressed the release of IL-2, IFN-γ, and TNF-α (Fig. 3C–E).

Up-regulation of immune checkpoint proteins can cause resistance to immune cell-mediated cytotoxicity in several cancer types. Among them, PD-L1 has received increasing attention in recent years^28^. We tested if PD-L1 could be upregulated under elevated pressure and cause resistance to αROR1-CAR T treatment. Again, we passaged cells under + 100 mmHg for 2 weeks, 1 week, 3 days, and 1 day and compared the expression of PD-L1 to cells cultured under normal pressure. As shown by our flow cytometry results (Fig. 3F,G), the expression of PD-L1 was upregulated by elevated pressure in a time-dependent manner and correlated with the cytotoxicity shown in Fig. 3A. We also measured another PD-1 ligand PD-L2 in A549 cells. Indeed, PD-L2 was also elevated in A549 cells (Fig. S1A). Interestingly, both PD-L1 and PD-L2 expression levels were increased by 100 mmHg in HepG2 liver cancer cells (Fig. S1B,C), suggesting that pressure could have suppressing effects on other cancer cells.

### PD-1 antibodies enhanced αROR1-CAR T killing of A549 lung cancer cells under elevated pressure

**Figure 4:**
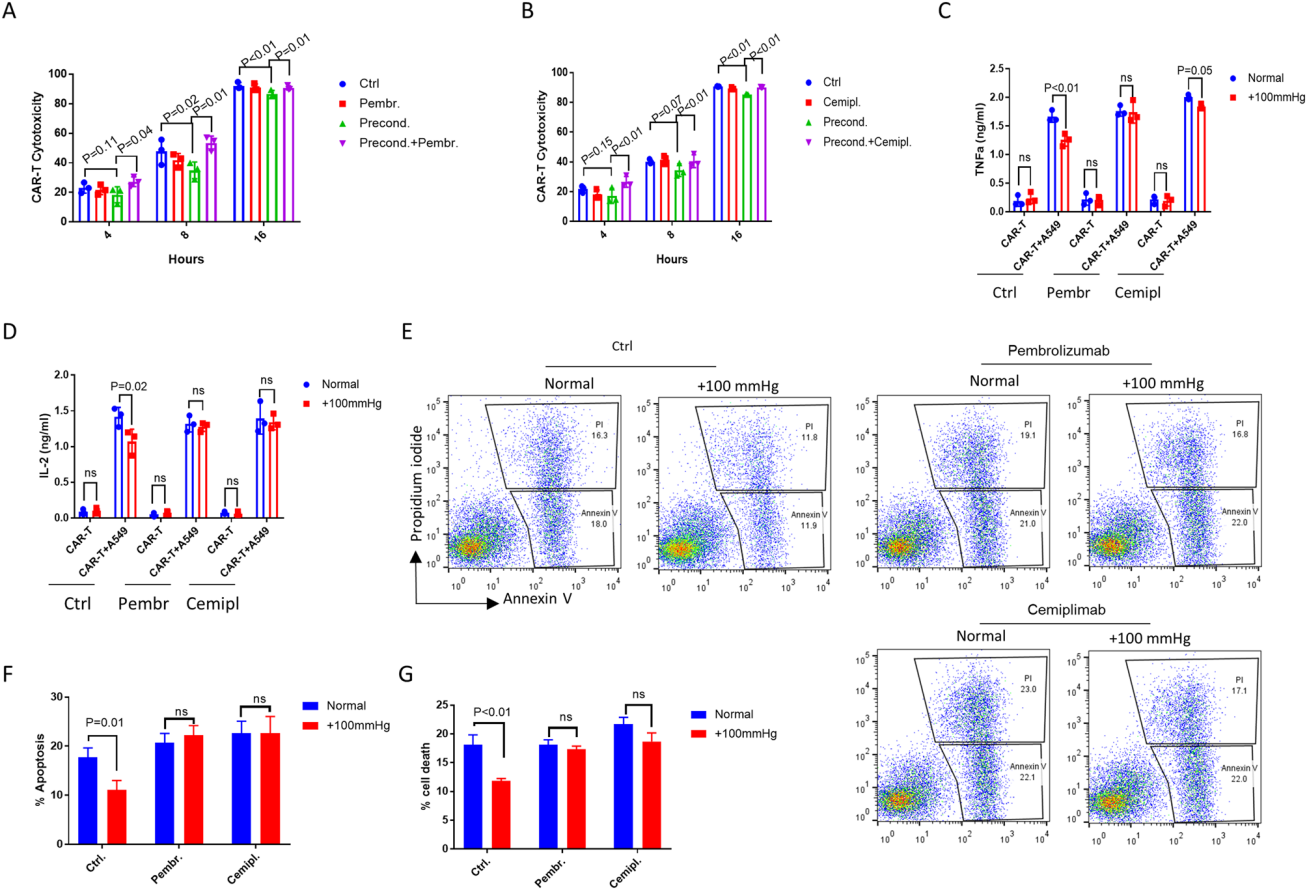
PD-1 antibodies enhanced αROR1-CAR T killing of A549 lung cancer cells under elevated pressure. (**A**,**B**) A549-Red-Fluc cells preconditioned in elevated pressure (+ 100 mmHg) for 7 days were incubated with non-preconditioned αROR1-CAR T cells under normal conditions. PD-L1 blocking antibodies Pembrolizumab (Pembr) and Cemiplimab (Cemipl) were added at 10 µg/ml at the same time. Cytotoxicity at indicated time points was calculated based on luciferase activity. (**C**,**D**) Cytokines (IFN-γ and TNF-α) in the medium of co-culture shown in (**A**) and (**B**) were measured by ELISA. (**E**) Flow cytometry analysis of co-cultures in (**A**) and (**B**) at 4 h. Annexin V indicates apoptosis and Propidium iodide indicates cell death. Shown are representative results of 3 biological repeats. (**F**,**G**) Quantification of apoptosis and cell death by 3 biological repeats of experiments in (**E**).

We asked if the increased expression of PD-L1/PD-L2 in A549 cells under elevated pressure could contribute to the decrease in CAR-T cell-mediated cytotoxicity. PD-L1/PD-L2 inhibits T cell cytotoxicity by interacting with PD-1 on T cell surface. The recent FDA-approved antibody drugs Pembrolizumab and Cemiplimab block such interaction, increase cancer antigen recognition, therefore contribute to cancer eradication. We tested if these antibodies could also mitigate the pressure-induced resistance to CAR-T cell treatment. By adding Pembrolizumab (10 µg/ml) or Cemiplimab (10 µg/ml) directly into the co-cultured αROR1-CAR T and A549 cells, we found that the pressure-induced resistance was greatly reduced (Fig. 4A,B). Consistently, the secretion of cytokines IFN-γ and TNF-α was largely rescued to the levels under normal conditions (Fig. 4C,D). In addition, the apoptotic cell death in pressure-preconditioned A549 cells was also robustly increased (Fig. 4E–G). Together, our data suggest that PD-L1 antibodies could enhance CAR-T-mediated cytotoxicity in solid tumors.

### Pressure preconditioning increased tumor size in A549 xenograft mice, which was blocked by Pembrolizumab

**Figure 5:**
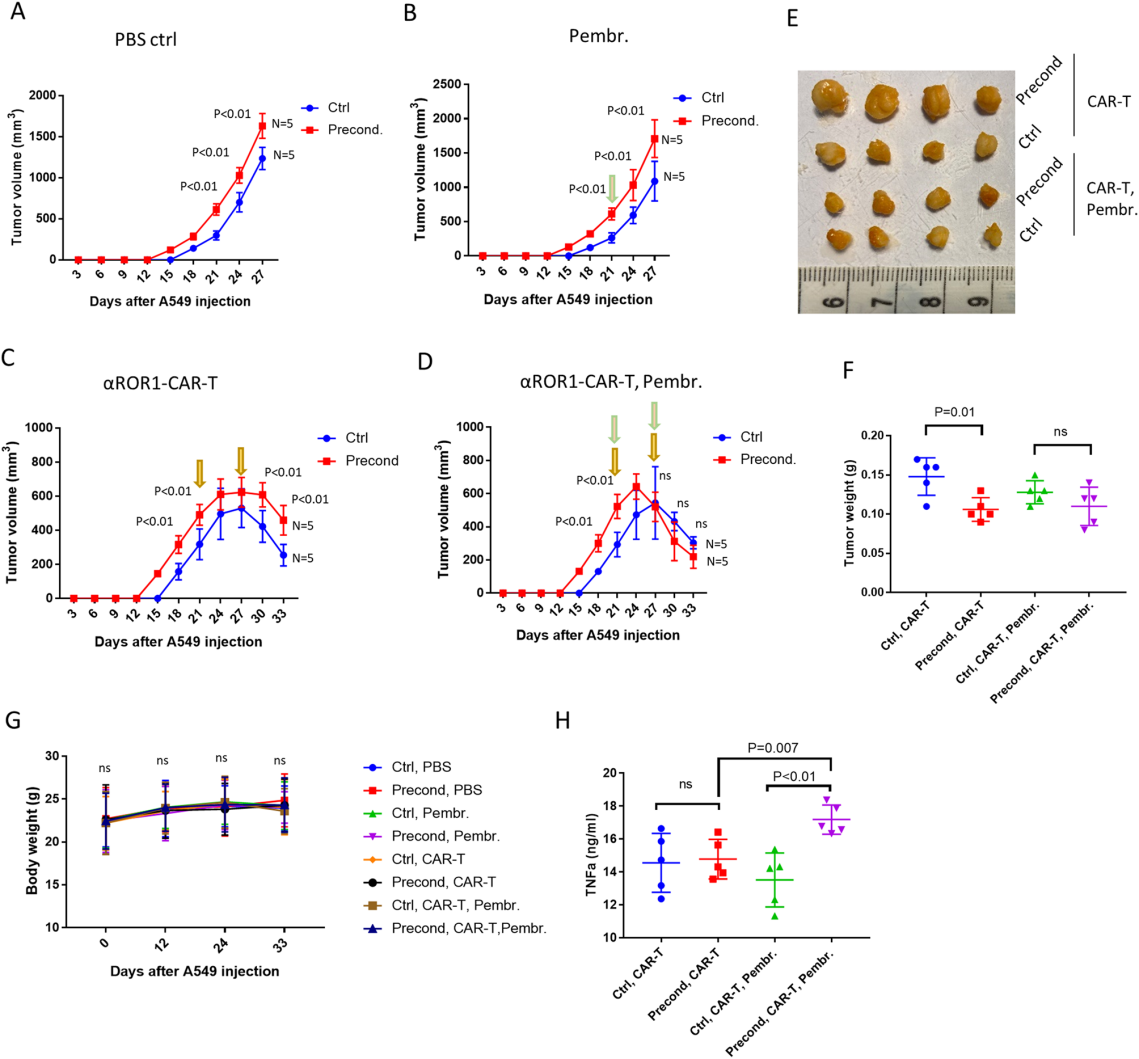
Pressure preconditioning increased tumor size in A549 xenograft mice, which was blocked by Pembrolizumab. (**A**) Pressure preconditioning increased tumor size in A549 xenograft mice. N indicates the number of mice and P indicates the P values by unpaired *t* test. A549 xenograft tumors were established subcutaneously in athymic nude mice and tumor size was examined every 3 days. (**B**) PD-L1 antibody Pembrolizumab did not affect tumor size in the absence of αROR1-CAR T cells. Pembrolizumab (5 mg/kg) was injected intravenously on day-21. (**C**) Pressure preconditioning increased tumor size in the presence of αROR1-CAR T cells. αROR1-CAR T cells were injected on day-21 and day-27. (**D**) Pembrolizumab enhanced the efficacy of αROR1-CAR T cells. Pembrolizumab (5 mg/kg) was injected intravenously on day-21 and day-27. Green arrows indicate adding Pembrolizumab and yellow CAR-T cells. (**E**) Tumors were surgically removed after sacrificing the mice on day-33 and imaged. (**F**) Tumors were weighed and statistically analyzed by unpaired *t* test. *ns* not significant. (**G**) CAR T cells and Pembrolizumab treatment did not affect the bodyweight of xenograft mice. (**H**) Plasma TNF-α levels were increased by combined treatment of CAR-T cells and Pembrolizumab.

To further confirm our in vitro study, we established xenograft tumors in male BALB/c athymic nude mice by injecting A549 cells subcutaneously. We followed the growth of the tumors and found that, interestingly, pressure-preconditioned A549 cells formed tumors faster than control A549 cells, resulting in a larger tumor size (Fig. 5A). Larger tumors were observed regardless of the PD-L1 antibody Pembrolizumab or αROR1-CAR T cells (Fig. 5B,C). However, when treated with Pembrolizumab and αROR1-CAR T cells in combination, pressure-preconditioned A549 tumors shrank faster, leading to similar tumor size after 6 days of treatment (Fig. 5D–F). All the treatments, including Pembrolizumab, αROR1-CAR T cells, or a combination did not change the weight of mice, nor did they change the appearance or mobility, suggesting a specific effect of the combined therapy (Fig. 5G). Consistently, the plasma TNF-α levels were increased specifically by the combined therapy of Pembrolizumab and αROR1-CAR T cells, but not by individual treatments (Fig. 5H). These results confirmed our in vitro study and suggested that PD-L1 checkpoint inhibitors could enhance CAR T cells cytotoxicity in solid tumors.

## Discussion

TIFP was initially thought to dampen chemotherapy simply by blocking drug delivery^32^. However, recent studies show that TIFP can actively modulate signaling transduction, cancerous proliferation, and metastasis^13–18^. Lowering TIFP has shown promising results in improving cancer chemotherapy^20,21^. However, compared to hypoxia and nutrient starvation, research on TIFP in vivo and in vitro remains scarce. In this study, by modeling TIFP in vitro, we show that elevated pressure (+ 100 mmHg) increased immune checkpoint PD-L1 expression, causing resistance to CAR T cell-mediated cytotoxicity. In addition, we show that the FDA-approved PD-1 blockage antibodies Pembrolizumab and Cemiplimab alleviated the pressure-induced resistance to CAR T cell therapy. In the xenograft mice, pressure preconditioning increases tumorigenesis, which was blocked by combining Pembrolizumab with CAR T cell therapy. Our study has provided novel knowledge regarding TIFP’s role in cancer progression, which could suggest strategies to better teat solid cancers.

The silence of CAR T and T cells in the tumor microenvironment is multilayers^7,33^. Our study has examined only the pressure effect in a short period. In our system, although PD-L1 expression in A549 cells played a predominant role in CAR-T cell resistance, it is unlikely that this mechanism would be accountable for all aspects of CAR T cells inhibition in solid tumors. In addition, although αROR1-CAR T cells were not affected by 24 h of elevated pressure in our system, most tumor infiltrating T cells reside in solid tumors for a longer time and likely would be affected by TIFP. Consistently with this idea, in the xenograft mice, PD-1 blocking antibody readily increased cytotoxicity of CAR T cells, indicating that these CAR T cells have been hampered to some degree in the pressured tumor microenvironment. In vitro*,* we found that PD-L1 expression was no longer increased over 14 days (Fig. 3F). If the advantage of pressure on tumor growth is simply due to PD-L1:PD-1checkpoint, we expect that the CAR-T and Pembrolizumab combination would remain reducing the tumors as shown in Fig. 5D. However, in the real tumor microenvironment, long-term pressure could involve additional mechanisms, which could dampen or even remove the effect of Pembrolizumab on shrinking pressured tumors. Change in ROR1 expression in cancer cells could also contribute to αROR1-CAR T cell cytotoxicity, however, our in vitro assay data showed that ROR1 expression was not repressed by TIFP in A549 cells (Fig. S2). Again, whether this is the case in the tumor microenvironment remains to be studied.

The infiltration of immune cells into solid tumors is limited. Decreasing TIFP is known to increase T cell infiltration and improve immunotherapy^20,21^. However, in our case, αROR1-CAR T is not likely functioning to decrease TIFP, because ROR1 is an immune checkpoint protein and has not been shown to decrease TIFP. In addition, our in vitro results show that αROR1 CAR-T eradiated A549 tumor cells effectively. Therefore, we believe that the infiltration of ROR1-CAR T cells into the solid tumors remains scarce. It is more likely that CAR-T cells are more active in combination with PD-1 antibodies, therefore contributing to improved cancer cell eradication. Based on the above reasons, strategies that reduce TIFP such as anti-VEGFR antibodies likely will increase αROR1-CAR T infiltration and further enhance cancer eradication.

Our study did not find any effect of TIFP on cytokine release and cytotoxicity CAR-T cells, consistent with a study in a hypertension mouse model^34^. In that study, increasing systolic pressure activated T cell, however, such pressure did not affect cytokine production in isolated human T cells and monocytes, nor did it affect the T-cell proliferation in these settings^34^. However, we cannot rule out the possibility that CAR-T cells are affected by pressure in vivo under the tumor microenvironment. The lack of activation of CAR-T cells by pressure in our study could simply be due to the ex vivo environment, where multiple cytokines required for T cell activation are missing. It would be interesting to study in vivo if CAR-T cells could be activated by elevated pressure either in mice models or human subjects.

Increasing PD-L1 and PD-L2 expression is a common strategy for cancer cells to evade immune surveillance^35^. Our findings of elevated PD-L1 and PD-L2 by pressure support several recent studies showing the matric stiffness in elevating PD-L1 expression in other cancer cells^36,37^. To disrupt the immune checkpoint PD1/PDL-1, we have been focused on PD-1 antibodies because PD-L1 antibody Atezolizumab showed a much weaker effect in enhancing CAR-T cytotoxicity in vitro. Also, due to the limited number of mice, we tested only Pembrolizumab that showed a more consistent effect on enhancing CAR-T cytotoxicity. At the dosage used in this study, we did not observe obvious changes in body weight (Fig. 5G) for different treatments, including the combination of CAR-T cells with checkpoint inhibitor Pembrolizumab. Neither did we observe any hints of cytokine release syndrome. However, we have not yet examined closely the serum levels of cytokines after the injection of CAR-T cells and Pembrolizumab. Therefore, a similar dosage might have adverse effects if applied in human subjects. It has been challenging to measure the TIFP in tumors because it varies depending on many factors including tumor types, size, location, and differentiation status. Different methods also result in several folds of difference. Available data show a wide range of TIFP from 5 to 40 mmHg in solid tumors^11^. However, in this range of pressure, we did not observe any change in either PD-L1 or PD-L2 expression in A549 cells (Fig. S3). Since the upper limit of TIFP has not been determined, the 100 mmHg pressure used in our study might still reflect the in vivo TIFP in certain tumors. However, care should be taken to interpret the data, as no in vitro model can fully reflect the complex in vivo system.

## Methods

### Cell culture

Human NSCLC cell line A549 stably expressing red firefly luciferase (A549-Red-Fluc) was purchased from PerkinElmer. A549 and HEK-293T cells are originally obtained from American Type Culture Collection. A549 and HEK-293T cells were maintained in Dulbecco's modified Eagle's medium (DMEM) supplemented with10% FBS. Fully anonymized human PBMCs were collected from healthy donors. Informed consent was obtained from all subjects and/or their legal guardian(s) and approval from the Ethics Committee of the Xiangya Hospital. PBMCs were cultured in AIM-V Medium (Gibco) containing 10% FBS and expanded by adding αCD3/αCD28 beads in a cell-to-bead ratio of 1:1 and IL-2 at 200 IU/ml. All cells were cultured in a humidified incubator with 5% CO_2_ at 37 °C. The pressure was applied using the Continuous Flow Constant Pressure cell culture (CFCPcc) device purchased from BioExcellence International Tech (Beijing, China). The device consists of an adjustable pressured pump connected to a sealed chamber in a normal CO_2_ incubator (Fig. S4). The pressure was controlled at 100 mmHg automatically by a sensor inserted in the cell culture chamber. The pump compresses the air in the incubator (74% N_2_, 21% O_2_, and 5% CO_2_) to the chamber continuously.

### CAR T cell generation

The αROR1-CAR was generated using the DNA fragment of scFv derived from a rabbit anti-human ROR1 mAb clone R12 published before^29^, cloned into CAR lentivirus backbone encoding a CD3ζ signaling domain and a 4-1BB co-stimulatory domain. The resulting αROR1-CAR-encoding lentivirus was produced via transient transduction of HEK-293T cells using Gibco LV-MAX Lentiviral Production system according to the manufacturer’s manual. Frozen human PBMCs from healthy donors were thawed in AIM-V Medium (Gibco) supplemented with 10% FBS, then activated by adding αCD3/αCD28 beads in a cell-to-bead ratio of 1:1 and IL-2 at 200 IU/ml. After 24 h, αROR1-CAR lentivirus was added to PBMCs. AIM-V Medium containing IL-2 was added every 2–3 days to dilute the growing PBMCs. PBMCs derived cells generally contain ~ 90% of T cells (CD4+ or CD8+) after 2 weeks of expanding (Fig. S5). The expression of CAR was confirmed by flow cytometry staining CAR-T cells with a recombinant ROR1 protein with human IgG Fc tag (Sino Biological), then a PE-conjugated antibody against human IgG Fc (R&D systems). The extraction of PBMCs were approved by Xiangya Hospital Ethics Committee (Reference number: 202106833) and all experiments were performed in accordance with guidelines and regulations of Xiangya Hospital Ethics Committee. For study involving human blood samples, all methods were carried out in accordance with institutional guidelines and regulations by Central South University. Informed consent was obtained from all subjects and/or their legal guardian(s).

### CAR T cell-mediated cytotoxicity assay

Cellular luciferase-based cytotoxicity was performed as shown before^38^. A549-Red-Fluc cells stably expressing red firefly luciferase were plated at the density of 20,000/well in a 96-well plate for 16 h. αROR1-CAR T cells at effector-to-target (E:T) ratios of 10:1 were added to A549-Red-Fluc cells. After 4, 8 and 16 h, A549-Red-Fluc cells on the plates were washed with PBS and 1 × cell lysis buffer was added directly to lyse cell by shaking at room temperature for 5 min. For each well, 50 μl cell lysate was transferred to white opaque plates and 50 μl luciferin working solution (Firefly Luc One-Step Glow Assay Kit, Pierce) was added. After incubation at room temperature for 10 min, luminescence was detected by SpectraMax Microplate Reader using a default protocol. CAR-T cytotoxicity was calculated by measuring the percentage of decrease in luminescence: 100 × (1 − (Luminescence of CAR-T-treated wells)/(Luminescence of non-treated cells)). The specificity of αROR1 scFv CAR T cells was confirmed by comparing to T cells transduced with an empty CAR vector, which showed only slight cytotoxicity towards A549 cells (Fig. S6).

### Cytokine release assay

After incubation of αROR1-CAR T cells with A549 cells for 16 h, cells on 96-well plates were centrifuged and supernatants were transferred to new plates. Cytokines released by CAR T cells were measured by using Human DuoSet ELISA kits according to the manufacturer’s protocol (R&D Systems). Cytokine concentrations were calculated by generating standard curves along with the experiments.

### Flow cytometry

CAR T cells were removed by washing the plates with cold PBS. A549 cells were trypsinized to detach from plates and washed with cold PBS pH7.4 containing 0.5% albumin, then incubated with primary antibodies or IgG isotype controls at suggested dilutions for 1 h. Cells were then washed extensively with (PBS, 0.5% albumin) and incubated with fluorochrome-conjugated secondary antibodies at suggested dilutions. For apoptosis, FTIC-Annexin V and Propidium iodide were added according to the manufacturer’s manual. Mouse monoclonal anti-human PD-L1 and PD-L2 antibodies (R&D Systems) were added to the cells at 200 × dilution and PE-conjugated goat anti-mouse secondary antibody (BioLegend) was added at 500 × dilution. For αROR1-CAR expression, ROR1 recombinant proteins were biotinylated and incubated with αROR1-CAR T cells for 1 h. After washing, PE-conjugated streptavidin was then added. Flow cytometry analysis was performed on cytoFLEX S (BECKMAN). The remaining CAR-T cells were further removed by size gating (Fig. S7). Data were analyzed and plotted by using the FlowJo (V10.7) software (https://www.flowjo.com/solutions/flowjo/downloads/previous-versions).

### Tumor xenograft model

Six-week-old male BALB/c athymic nude mice were obtained from Hunan Fenghui Biotechnology Co., Ltd. Animal experiments were approved by Animal Care and Use Committee of Xiangya Hospital (Reference number:202106833). Control or pressure-conditioned (+ 100 mmHg for 14 days) A549 cells (1 × 10^7^ in 0.1 ml PBS) were injected subcutaneously into the right-lower flank of the nude mice (20 controls and 20 pressure-conditioned). Each group was further divided into 4 subgroups (5 animals/group) for 4 treatments: PBS, αROR1-CAR T cells, Pembrolizumab, and αROR1-CAR T cells plus Pembrolizumab. The tumors were measured every 3 days using a Vernier caliper and tumor volume calculated as follows: tumor volume (mm^3^) = (shorter diameter^2^ × longer diameter)/2. CAR-T cells (1 × 10^7^ cells/mice) or/and Pembrolizumab (5 mg/kg) were injected intravenously 21 and 27 days after injection of A549 cells. At the end of the experiment, mice were sacrificed and tumors were surgically removed and imaged. In addition, plasma was collected from the blood for ELISA on human IFN-γ. All methods involved in the animal study are reported and carried out in accordance with ARRIVE guidelines. For mice study, all methods were carried out in accordance with institutional guidelines by Central South University.

### Statistical analysis

Data were plotted and analyzed by using GraphPad Prism software version 7.04 (https://www.graphpad.com/support/prism-7-updates/). All experiments contain at least 3 biological repeats. The difference between the mean values was analyzed by paired Student’s *t* test if not otherwise indicated. P values less than 0.05 were considered statistically significant in this study.

## Supplementary Information

Supplementary Figures.

## Data Availability

The αROR1-CAR DNA sequence was deposited in GeneBank (accession number OM468897). All other data used to support the findings of this study are included within the article.
