# Supplementary material for: Pressure increases PD-L1 expression in A549 lung adenocarcinoma cells and causes resistance to anti-ROR1 CAR T cell-mediated cytotoxicity
Source: Sci Rep. 2022 Apr 28;12:6919. doi: 10.1038/s41598-022-10905-6 (PMC9051206; doi:10.1038/s41598-022-10905-6)
Supplement: Supplementary file 1 — Supplementary Figures. [file 41598_2022_10905_MOESM1_ESM.docx]

Pressure increases PD-L1 expression in A549 lung adenocarcinoma cells

and causes resistance to anti-ROR1 CAR T cell-mediated cytotoxicity Zhenglin Ou1,2, Xiaolin Dou1,2, Neng Tang1,2^*^, Guodong Liu1,2,3*

Supplemental Figures


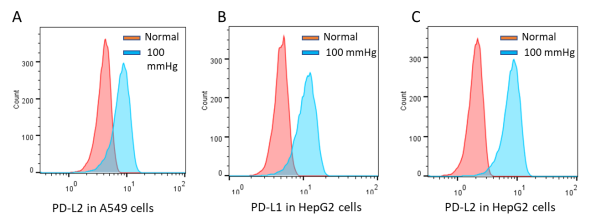


**Fig** **S1.** **PD-L1** **and** **PD-L2** **expression** **elevated** **in** **HepG2** **cells** **and** **A549** **cells.** (A) Expression

PD-L2 in A549 was elevated by 100 mmHg. (B-C) Expression ofPD-L1 and PD-L2 was elevated in HepG2 hepatocellular carcinoma cells. PD-L1 and PD-L2 expression were detected by specific antibodies and quantified by using flow cytometry. Data were presented in histogram by using Flowjo software.


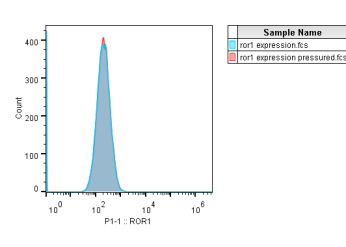


**Fig** **S2.** **ROR1** **expression** **was** **not** **repressed** **by** **TIFP** **in** **A549** **cells.** A549 cells were cultured under 100 mmHg pressure for 7-days and expression ofROR1 were detected by an specific antibody against extracelluar domain ofROR1 in flow cytometry. Shown are representative data of N>3 experimental repeats.


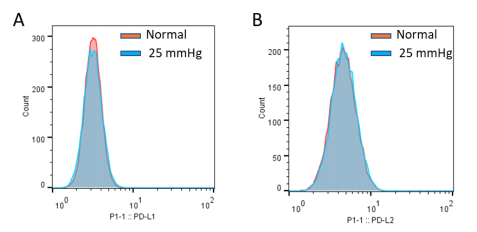


**Fig** **S3.** **Pressure** **at** **25** **mmHg** **did** **not** **affect** **PD-L1** **(A)** **and** **PD-L2** **(B)** **expression.** PD-L1 and PD-L2 expression were detected by specific antibodies and quantified by using flow cytometry. Data were presented in histogram by using Flowjo software.


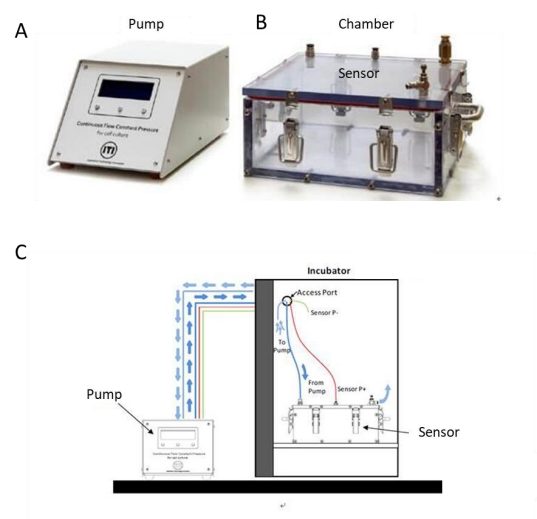


**Fig** **S4.** **Pressure** **control** **system.** (A) Sensor-controlled pump (control unit). (B) Pressured chamber (Cell culture vessel). (C) The working mechanism. The pump continuously pumps air from the incubator into the pressurized chamber in the incubator (blue line). A sensor is inserted in the chamber, which serves to automatically switch on/off the pump (line).


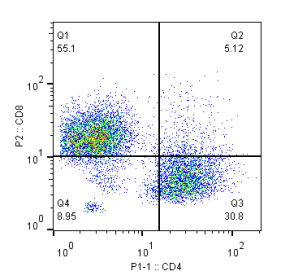


**Fig** **S5.** **PBMCs** **derived** **cells** **generally** **contain** **~90%** **of** **T** **cells** **(CD4+** **or** **CD8+)** **after** **2** **weeks** **of** **expanding**. PBMCs from healthy donors were thawed in AIM-V™ Medium (Gibco) supplemented with 10% FBS, then activated by adding αCD3/αCD28 beads in a cell-to-bead ratio of 1:1 and IL-2 at 200 IU/ml. After 24 h, αROR1-CAR lentivirus was added to PBMCs. AIM-V™ Medium containing IL-2 was added every 2–3 days to dilute the growing PBMCs. T cell subpopulation were analyzed by flow cytometry. Shown are representative data of N>3 biological repeats.


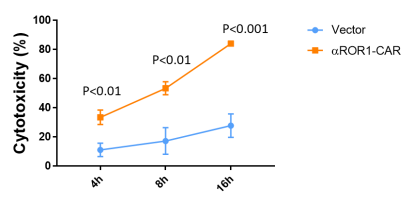


**Fig** **S6.** **The** **specificity** **of** **αROR1** **scFv** **CAR** **T** **cells.** PBMCs transduced and with αROR1

CAR or an empty CAR vector were co-cultured with luciferase-expressing A549 cells for indicated time and cytotoxicity were measured with Firefly Luc One-Step Glow Assay Kit. Shown are average of N = 3 biological repeats. Error bars indicated standard deviation ofthe mean.


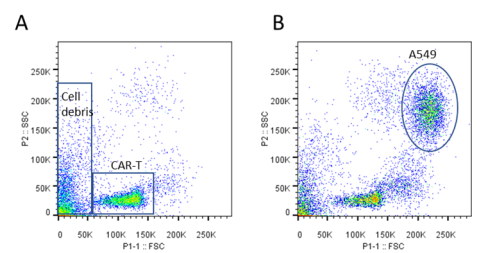


**Fig** **S7.** **Size** **gating** **to** **remove** **remaining** **T** **cells** **from** **A549** **cancer** **cells.** T cells were strongly attached to A549 cells and were not readily removed by washing with PBS. To further remove the attaching T cells, A549 cells were trypsinized and gated through size difference in flow cytometry analysis. (A) CAR T cells only control. (B) A549 cells from CAR T cytotoxicity assay. FSC, forward scattering. SSC, side scattering.
